# Comparison of gepant effects at therapeutic plasma concentrations: connecting pharmacodynamics and pharmacokinetics

**DOI:** 10.1186/s10194-024-01846-8

**Published:** 2024-08-28

**Authors:** Deirdre M. Boucherie, Ruben Dammers, Arnaud Vincent, A. H. Jan Danser, Antoinette MaassenVanDenBrink

**Affiliations:** 1 Division of Pharmacology and Vascular Medicine, Department of Internal Medicine, Erasmus MC University Medical Center, Rotterdam, the Netherlands; 2 Department of Neurosurgery, Erasmus MC University Medical Center, Rotterdam, the Netherlands

**Keywords:** Anti-CGRP, Atogepant, Gepant, Migraine, Rimegepant, Trigeminal, Ubrogepant, Zavegepant

## Abstract

**Background:**

Orally administered second-generation gepants are effective for the treatment of migraine. The intranasal administration of the third-generation gepant zavegepant might have additional benefits including a rapid onset of action, but it is not clear yet to which extent this has clinical relevance.

**Methods:**

We examined the effect of zavegepant on the relaxations induced by calcitonin gene-related peptide (CGRP) in human isolated middle meningeal arteries. Furthermore, we connected the pharmacodynamics and pharmacokinetics of gepants by combining data from clinical and basic research.

**Results:**

We showed that 10 nM zavegepant potently antagonized the functional response to CGRP. We also showed that all gepants are effective at inhibiting functional responses to CGRP at their therapeutic plasma concentrations.

**Conclusions:**

The relatively low predicted potency of zavegepant to inhibit CGRP-induced relaxation at therapeutic systemic plasma concentrations may point to the relevance of local delivery to the trigeminovascular system through intranasal administration. This approach may have additional benefits for various groups of patients, including overweight patients.

**Supplementary Information:**

The online version contains supplementary material available at 10.1186/s10194-024-01846-8.

## Background

Calcitonin gene-related peptide (CGRP) plays a crucial role in the pathophysiology of migraine. During a migraine attack, the levels of the potent vasodilatory neuropeptide CGRP are elevated, and infusion of CGRP may induce migraine-like attacks in patients with migraine [25, 26, 30, 37]. Upon stimulation of the trigeminal ganglion, CGRP is released from trigeminal neurons, thereby activating nociceptive pain pathways and inducing intracranial vasodilation [25].

This knowledge on the role of CGRP in the pathogenesis of migraine has led to the development of drugs directed against CGRP and its receptor, i.e., CGRP(-receptor) targeting monoclonal antibodies and small-molecule CGRP receptor antagonists called gepants. In the early twenty-first century, several small-molecule CGRP receptor antagonists were developed, the majority of which had moderate affinity for the CGRP receptor [42]. These discoveries paved the way for the first generation of gepants that were advanced into clinical trials, namely olcegepant and telcagepant. Although the first-generation gepants were effective for the acute treatment of migraine attacks, their development was ceased. Telcagepant induced liver toxicity [33], whereas the low bioavailability of olcegepant and, therefore, its intravenous administration were considered a clinical limitation [45, 49]. The second-generation gepants ubrogepant, rimegepant, and atogepant are administered orally and were developed for either the acute treatment of attacks (ubrogepant), preventive treatment (atogepant), or both (rimegepant). These gepants are effective and do not exhibit any liver toxicity [50]. All three second-generation gepants have received approval from the FDA and EMA.

The third-generation gepant zavegepant (formerly named vazegepant) was developed for the acute treatment of migraine as an intranasal formulation. Intranasal administration can induce rapid effects as a drug can move across a single layer of epithelial cells into the bloodstream, leading to several benefits over oral formulations including a shorter time to reach the maximum plasma concentration and, thus, a faster onset of treatment effects and rapid pain relief [16, 46]. Additionally, intranasal delivery could potentially provide advantages for patients with severe nausea or vomiting [38]. Finally, it cannot be excluded that intranasal delivery would exert a direct trigeminovascular effect [23, 35, 41], although we are not aware of any data that could substantiate or refute this hypothesis. Zavegepant is currently in late-stage development and appears both efficacious and safe, also in terms of hepatotoxicity [16, 38]. Whether an intranasal formulation is more efficacious than oral administration and whether all gepants are equally efficacious and safe remain to be determined, as head-to-head trials for gepants have not been performed. Moreover, information on which group of patients would benefit from which gepant or administration method is highly relevant as gepants enter the market.

Our group has previously studied and compared the vascular effects of the then available gepants in coronary and intracranial arteries in a preclinical setting [18, 21, 27, 28, 43, 47]. In the current study, we aimed to characterize the functional response to CGRP in the absence or presence of the newest gepant zavegepant in human middle meningeal arteries (HMMA). With the middle meningeal artery being innervated by trigeminal nerve endings, this vascular bed is a relevant proxy for functional responses of the trigeminovascular system. Our second aim was to compare the blocking effect of gepants at therapeutic concentrations on CGRP-induced relaxation calculated from clinical gepant plasma levels. If the systemic concentrations of gepants are related to efficacy, which we assume to be similar across all gepants [19, 29, 40], we would expect a similar blocking effect towards CGRP for all gepants. The method utilized here enables the connection of in vitro pharmacodynamics to clinical pharmacokinetics.

## Methods

### Characterisation of zavegepant in human isolated middle meningeal arteries

HMMAs were obtained from 6 patients (2 females, 4 males; 52 ± 19 years of age) undergoing neurosurgery at Erasmus MC University Medical Center, Rotterdam, the Netherlands. All patients that were planned for pterional or temporal approaches indicated for tumour surgery, aneurysm surgery, or trauma surgery were eligible for HMMA harvesting. HMMAs were harvested by excision from the dura only if they did not experience any trauma by the craniotomy. Immediately upon dissection, the HMMAs were stored at 4 °C in Medium 199 (Westburg, the Netherlands) and rapidly transported to the laboratory. The surrounding dura and connective tissue were gently removed and the HMMA was stored overnight at 4 °C in oxygenated (95% O_2_, 5% CO_2_) high-glucose Krebs buffer (119 mM NaCl, 4.7 mM KCl, 1.25 mM CaCl_2_, 1.2 mM MgSO_4_, 1.2 mM KH_2_PO_4_, 25 mM NaHCO_3_, and 11.1 mM glucose; pH = 7.4).

For functional measurements the arteries were cut into 2-mm segments and mounted using Ø 40 μm stainless-steel wires in Mulvany myograph organ baths (Danish Myo Technology, Aarhus, Denmark) filled with high-glucose oxygenated Krebs buffer at 37 °C. Tension data were recorded using LabChart data acquisition (AD Instruments, Oxford, UK). After 30 min of equilibration, the tension of the arteries was normalized to 90% of the estimated diameter at 100 mmHg transmural pressure [44]. To assess viability and to obtain an internal standard for the contractile capacity per segment, the segments were contracted with 30 mM KCl, washed, and subsequently contracted with 100 mM KCl. Next, the segments were incubated with or without 10 nM zavegepant (zavegepant hydrochloride HY-132131, Bio-Connect, the Netherlands) dissolved in dimethyl sulfoxide and diluted in milliQ for 30 min prior to constructing a concentration–response curve for human αCGRP (Polypeptide Group, Baar, Switzerland; 0.01 nM – 1 µM, half logarithmic steps). After thorough washing, endothelial function was assessed with substance P (MCE, HY-P0201; 10 – 100 nM) after precontraction with U46619 (Sigma-Aldrich, D8174; 10 – 100 nM). CGRP-induced relaxation was expressed as a percentage of the precontraction to 30 mM KCl. Using nonlinear regression in Prism 8 (GraphPad Software, San Diego, CA, USA), pEC_50_ values and the pK_B_ were calculated for each experiment to assess potency. In case that the concentration–response curve with zavegepant did not reach a plateau, the pEC_50_ was interpolated from a nonlinear regression that was restricted to the E_max_ obtained with the corresponding control segment. A paired t-test was performed for pEC_50_ values and KCl responses.

### Analysis of gepant potency

#### Data collection

We collected data from the literature for the following gepants: rimegepant, ubrogepant, atogepant, and zavegepant, all of which were previously or currently in clinical development. Data on clinical pharmacokinetics, plasma-protein binding, and the (proposed) clinical dose were collected. Pharmacodynamic data on the potency of the aforementioned gepants, excluding zavegepant, were sourced from prior studies conducted within the same laboratory at the Erasmus MC University Medical Center in Rotterdam, the Netherlands using the same methodology [27, 28, 43, 47]. The original data on the pharmacological characterization of zavegepant are presented in the current paper.

#### Analysis of pharmacokinetic data

**Fig. 1:**
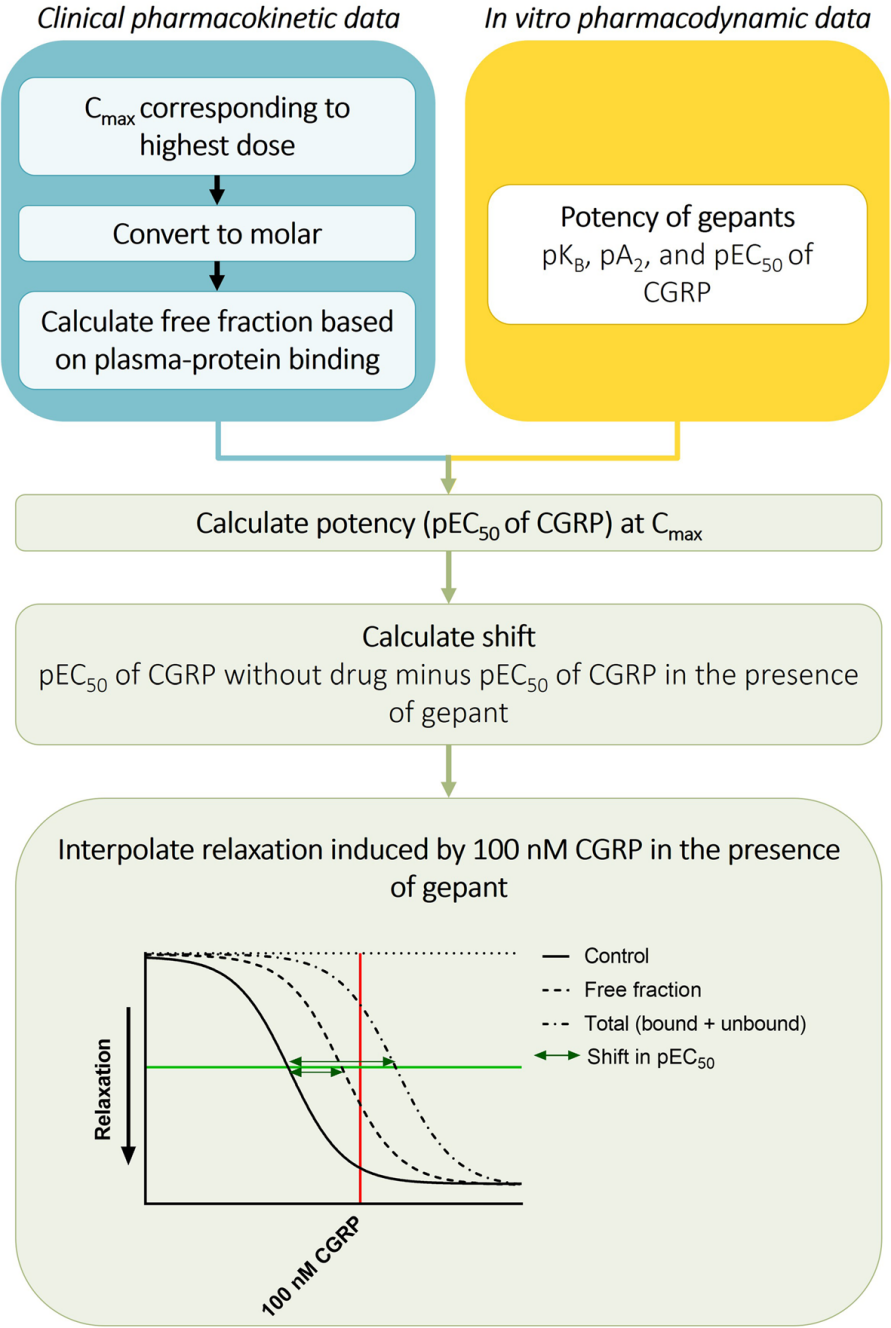
Schematic overview of calculations of the potency of the gepants at their therapeutic plasma concentrations

First, we transformed the data on therapeutic plasma concentrations of gepants and calculated total and plasma protein-corrected values. For a schematic overview of the analysis, see Fig. 1. Pharmacokinetic data (i.e., C_max_) from the highest approved dose were used, because these data were accessible for all gepants and this approach would thus enhance comparability. C_max_ values were first converted to nanomolar concentrations to facilitate comparing pharmacokinetic data between the gepants. Next, C_max_ values were corrected for plasma-protein binding to obtain the free fraction C_max_. For subsequent calculations, we used both the values of the total C_max_ (bound and unbound) and the free fraction C_max_ (corrected for plasma-protein binding), as a small deviation in the amount of plasma-protein binding affects the free fraction to a major extent, and we considered therefore the comparison between data more balanced when presenting both total and plasma protein-corrected values.

#### Connecting pharmacodynamics and pharmacokinetics

We then calculated the potency of the gepants at these plasma concentrations to block CGRP-induced relaxation (see Fig. 1). To this end, we combined the abovementioned pharmacokinetic data with pharmacodynamic data on the potency of gepants (i.e., pEC_50_, pK_B_, or pA_2_ values, whichever were available). We used potency data obtained with 10 nM of each gepant. The pharmacodynamic data were used to calculate the pEC_50_ values that CGRP would have in the presence of gepant levels corresponding to the C_max._ The pEC_50_ of CGRP in the absence of an antagonist served as a control. Subsequently, we interpolated relaxation that would occur in response to 100 nM CGRP in the presence of the C_max_. To achieve this, the shift to the right was calculated by subtracting the calculated pEC_50_ from the control pEC_50_. The concentration of 100 nM CGRP was used in the analysis because this concentration induces almost the maximum relaxation in the absence of a gepant and was included in all pharmacological characterizations. To enhance the accuracy of the interpolation, our analysis focused solely on gepants with C_max_ values and pharmacological characterizations within the nanomolar range, thereby excluding telcagepant and olcegepant. This approach reduced the need for assumptions, particularly considering that Schild plot data in HMMA were not available for all gepants.

## Results

### Functional responses of the human middle meningeal artery to CGRP in the absence or presence of zavegepant

**Fig. 2:**
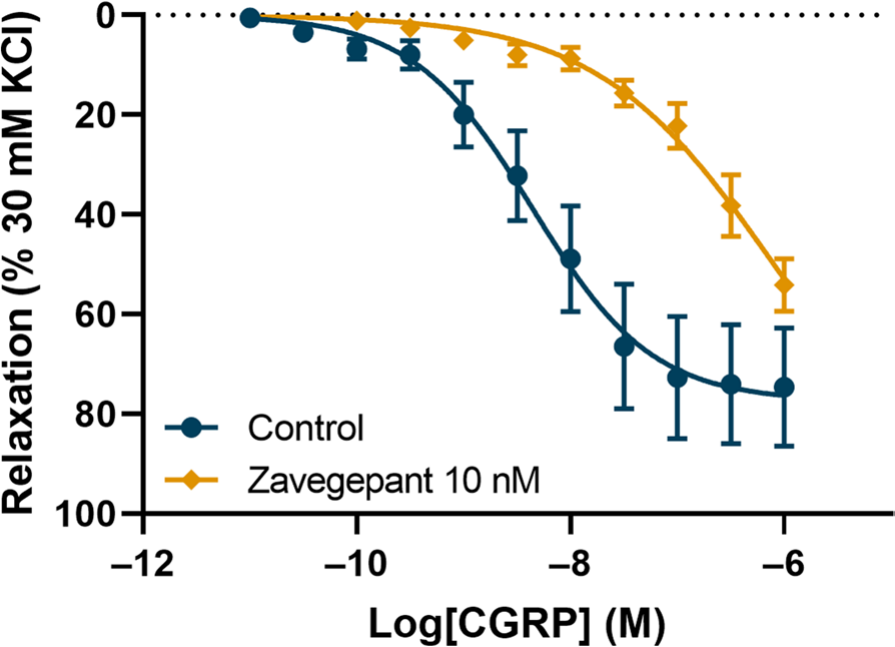
The effect of 10 nM zavegepant on the αCGRP-induced relaxation of human middle meningeal arteries (*n* = 6). The results are expressed as the mean ± SEM

We aimed to characterise the functional response to human αCGRP in the absence or presence of 10 nM zavegepant in HMMA. Prior to the construction of the concentration–response curve to CGRP, the HMMA segments were challenged with 30 mM KCl and 100 mM KCl, which did not differ between the groups (30 mM KCl: control 6.92 ± 5.11 mN, zavegepant 7.47 ± 3.44 mN; KCl 100 mM: control 5.49 ± 4.99 mN, zavegepant 5.82 ± 3.17 mN). All HMMA segments had functional endothelium (substance P-induced relaxation: control 72.3 ± 6.2% of precontraction obtained with U46619, zavegepant 62.8 ± 32.9%). CGRP induced a functional vasorelaxant response with an E_max_ of 75.1 ± 11.6%. Zavegepant at 10 nM significantly shifted the concentration–response curve to CGRP to the right (control pEC_50_: 8.40 ± 0.09; 10 nM zavegepant pEC_50_: 6.38 ± 0.07; *p* = 0.0001; mean ± SEM; Fig. 2). Zavegepant did not induce vasocontraction. The pK_B_ for 10 nM zavegepant was 10.02 ± 0.07. The pA_2_ value could not be calculated because we used only one concentration of zavegepant.

### Comparison of the potency of gepants at therapeutic concentrations to block CGRP-induced relaxation

**Fig. 3:**
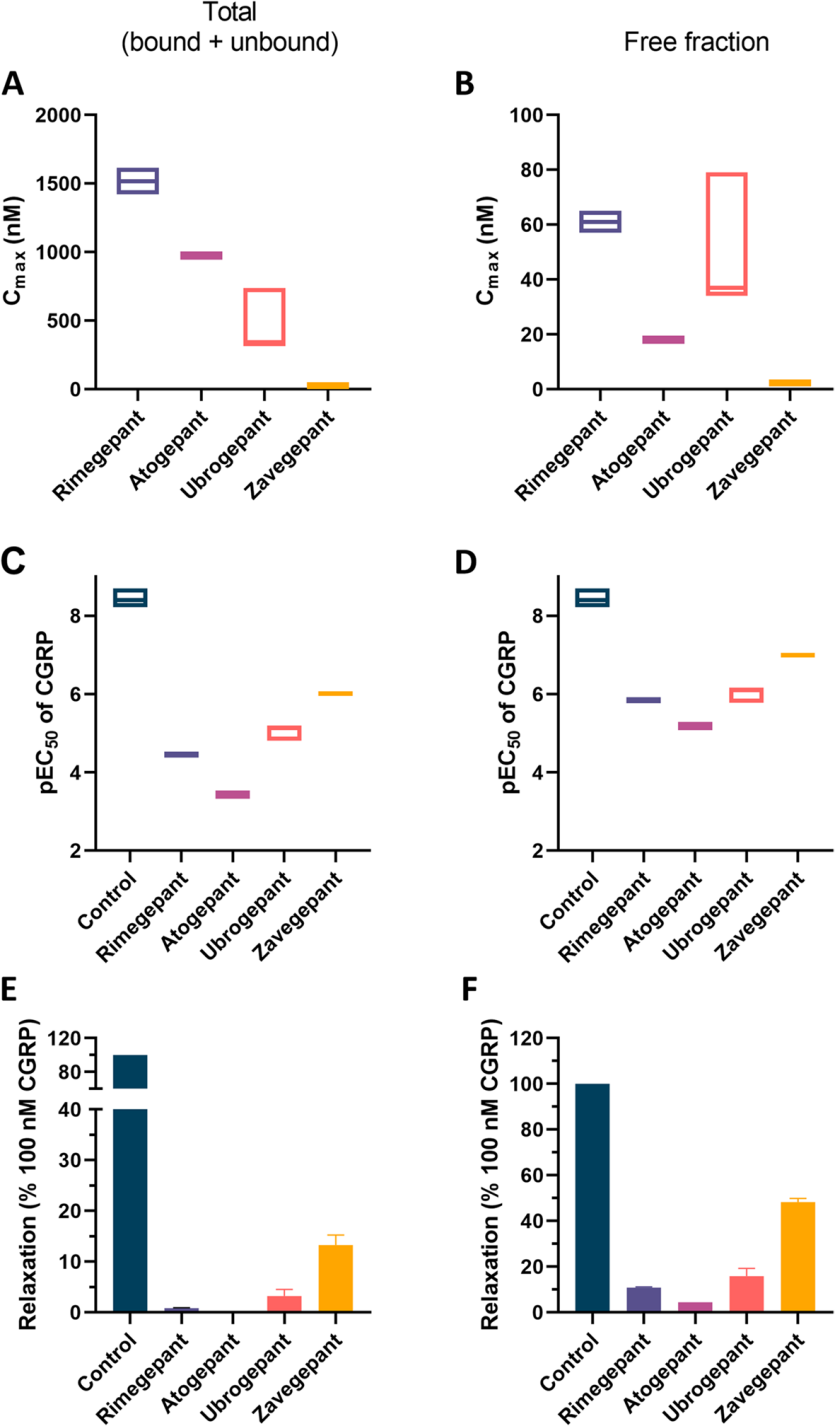
Gepant plasma concentrations, pEC_50_ of CGRP, and interpolated CGRP-induced relaxation in the absence or presence of therapeutic gepant plasma concentrations, based on values of total C_max_ (bound and unbound; A, C, E) and free fraction C_max_ (corrected for plasma-protein binding; B, D, F). A-B) Maximum plasma concentration (C_max_) measured in clinical trials. C-D) pEC_50_ of CGRP in the absence or presence of gepants at their therapeutic plasma concentrations (C_max_). E–F) Relaxation induced by 100 nM CGRP in the presence of a therapeutic concentration of the gepants, as a percentage of the relaxation induced by 100 nM CGRP in the absence of a gepant. All data are presented as median with range

In the second part of our analysis, we calculated the potency of gepants at their therapeutic plasma concentrations to block relaxation induced by 100 nM CGRP. To this end, we performed calculations based on both clinical pharmacokinetics and in vitro pharmacodynamics (see Supplemental Table 1). Concerning the clinical pharmacokinetics, the median total C_max_ ranged from 24 nM for zavegepant to 1,420 nM for rimegepant and the median free fraction C_max_ ranged from a median of 2.4 nM (zavegepant) to 57 nM (rimegepant) (Table 1; Fig. 3A-B).

**Table 1:** Calculated pEC_50_ of CGRP and clinical pharmacokinetics used for calculations

Gepant	Clinical dose	MW (g/mol)	Plasma-protein binding	Free fraction	Reported C_max_	*Total*		*Free fraction*	
						**C**_**max**_** (nM)**	**Calculated pEC**_**50**_** of CGRP**	**C**_**max**_ **(>nM)**	**Calculated pEC**_**50**_** of CGRP**
Rimegepant	75 mg, oral^a^	534.56 (or 610.63^d^)	96% [10]	4%	75 mg: 759.2 ± 23.0 ng/mL (mean ± SD)^b^ [6]	1,420	4.48	56.8	5.87
					75 mg: 862.9 ng/mL (43) (GM, CV%) [8]	1,614	4.42	64.5	5.82
Atogepant	10, 30, 60 mg, oral	603.5	98.2% [11]	1.8%	60 mg: 589 ± 248 ng/mL (mean ± SD)^b^ [11]	976	3.43	17.6	5.18
Ubrogepant	50 or 100 mg, oral	549.5	89.3% [12]	10.7%	100 mg: 344, 241–491 (median, 95% CI)^b^ [4]	344	5.14	36.8	6.11
					100 mg: 316, 250–400 nM (median, 95% CI)^c^ [4]	316	5.18	33.8	6.15
					100 mg: 405.76 ± 218.9 ng/mL (mean ± SD)^b^ [12]	738	4.81	79.0	5.78
Zavegepant	10 mg, intranasal	638.8 (or 675.28^d^)	90% [24]	10%	10 mg: 13.40 ng/mL (52.87) (GM, CV%)^b^ [9]	21	6.06	2.1	7.04
					10 mg: 16.31 ng/mL (72.06) (GM, CV%)^c^ [9]	26	5.97	2.6	6.95
*CGRP* *(Control)*	*-*		*-*		*-*	*-*	*8.4 (8.2 – 8.7)*	*-*	*8.4 (8.2 – 8.7)*

*CI* Confidence interval, *C*_*max*_ maximum plasma concentration, *CV* Coefficient of variation, *GM* Geometric mean, *MW* molecular weight, *pEC*_*50*_ negative log concentration of CGRP that would induce half of the maximum response in the presence of the gepant, *SD* Standard deviation

^a^75 mg per day (acute). Dose used in phase 2/3 clinical trials for prophylaxis: 75 mg every other day

^b^Based on a group receiving a single dose

^c^Based on day 1 of a group receiving QD treatment

^d^In case of the form with sulphate attached

Next, we used the C_max_ to calculate the potency of each gepant to block CGRP-induced relaxation at their therapeutic concentrations by means of the pEC_50._ While the median pEC_50_ of CGRP in the absence of a gepant was 8.4 (range 8.2–8.7), the median pEC_50_ of CGRP in the presence of a gepant ranged from 3.4 (atogepant) to 6.0 (zavegepant) for the total C_max_ and from 5.2 (atogepant) to 7.0 (zavegepant) for the free fraction C_max_ (Table 1; Fig. 3C-D), demonstrating a clear shift to the right for all gepants at therapeutic concentrations.

Then, we interpolated CGRP-induced relaxation when a gepant at a therapeutic concentration (i.e., C_max_) would be present. With the response to 100 nM CGRP in the absence of a gepant set to 100%, all gepants were able to block CGRP-induced vasorelaxation (Fig. 3E-F). Atogepant was most potent for both the calculations based on total C_max_ and free fraction C_max_: 100 nM CGRP would induce 0% (total C_max_) or 4.5% (free fraction C_max_) relaxation in the presence of atogepant, followed by rimegepant (0.9% and 12.4%), ubrogepant (3.2% and 18.6%), and zavegepant (13.2% and 48.2%).

### Demographics and clinical characteristics of the trial populations

Clinical pharmacokinetic data were mainly obtained from small samples of healthy volunteers, with the exception of rimegepant, which was measured both in healthy volunteers and lactating women (Table 2). The age of the participants, sex distribution, and body mass index were not reported for any of the studies.

**Table 2:** Demographics and clinical characteristics of the trial populations included in our analysis

Gepant	Trial population	n	Women (%)	Age (years)	BMI (kg/m^2^)	Genetic background	Reference
Rimegepant	Lactating women	12	100	29.8 ± 3.6^a^	26.8 ± 4.9^a^	*Race:* White, 83.3% American Indian or Alaska Native, 8.3% Multiple, 8.3% *Ethnicity:* Hispanic or Latino, 25.0%	[6]
	Healthy volunteers	6	50	41.5 ± 6.6^a^	28.7 ± 1.9^a^	*Race:* White, 100% *Ethnicity:* Hispanic or Latino, 83.3%	[8]
Atogepant	Healthy volunteers	8	37	58.4 ± 3.2^a^ (55–63)^b^	28.8 ± 2.8^a^ (24.9–32.8)^b^	*Race:* White, 87.5% Black/African American, 12.5% *Ethnicity:* Hispanic or Latino, 63%	[11]
Ubrogepant	Healthy volunteers	6	0	21–51^b^	18– ≤ 32	*Race:* White, 100% *Ethnicity:* Hispanic or Latino, 0%	[4]
	Healthy volunteers	8	50	58.1 ± 2.8^a^ (54–61)^b^	28.3 ± 2.4^a^	*Race:* White, 100% *Ethnicity:* Hispanic or Latino, 62.5%	[12]
Zavegepant	Healthy volunteers	8	NA	18–55^b^	18.5–30.0^b^	NA	[9]

*NA* Not available

^a^Mean ± SD

^b^range

## Discussion

Our first objective was to characterize the response to CGRP in the absence or presence of zavegepant, the only intranasally administered gepant, in HMMAs. Our results demonstrated that 10 nM zavegepant potently antagonizes the functional response to CGRP. Our second aim was to compare the blocking effect of therapeutic gepant concentrations on CGRP-induced relaxation. Our comparison revealed that all approved gepants effectively block the response to CGRP at their respective therapeutic plasma concentrations, with atogepant exhibiting the most potent inhibitory effect and zavegepant demonstrating the least inhibitory effect.

Although no head-to-head trials have been performed, several meta-analyses of gepants have been published recently that corroborate the assumption of similar efficacy across gepants. For the preventive gepants atogepant and rimegepant, the primary endpoint of reduction in mean monthly migraine days compared with placebo varied between -0.8 [-1.56; -0.04] (OR [95% CI]) for rimegepant 75 mg and -1.35 [-1.85; -0.85] for atogepant 60 mg [29]. For the acutely acting gepants rimegepant, ubrogepant, and zavegepant, two-hour pain relief versus placebo was between 1.16 [1.06–1.24] (RR [95% CI]) for zavegepant 10 mg and 1.37 [1.27–1.46] for rimegepant 75 mg in a meta-analysis [19]. In a preliminary meta-analysis of rimegepant and zavegepant, the percentage of patients experiencing two-hour pain relief was very similar for rimegepant 75 mg and zavegepant 10 mg (58.0% [56.0–60.2] versus 59.5% [56.4–62.5]) [40]. No comparison was available with data on two-hour pain freedom of the acute gepants. Intranasal delivery facilitates rapid absorption through the nasal mucosa, which leads to a fast onset of action, and might also make it suitable for patients experiencing nausea and vomiting. However, based on the similar efficacy observed among gepants, it is still debated whether the intranasal delivery of zavegepant offers additional therapeutic advantages compared to the orally administered gepants [19, 29, 38, 40, 46]. Notably, comparing clinical efficacy does not take into account that zavegepant exhibits a lower systemic concentration compared to the other gepants. These lower concentrations are confirmed by a lower blocking effect of CGRP in our analyses. If systemic concentrations were linked to efficacy, the therapeutic efficacy of zavegepant should have been inferior to that of the orally administered gepants. Given the fact that the efficacy is similar across gepants, we propose that the intranasal formulation of zavegepant could offer several benefits when compared to orally administered gepants.

**Fig. 4:**
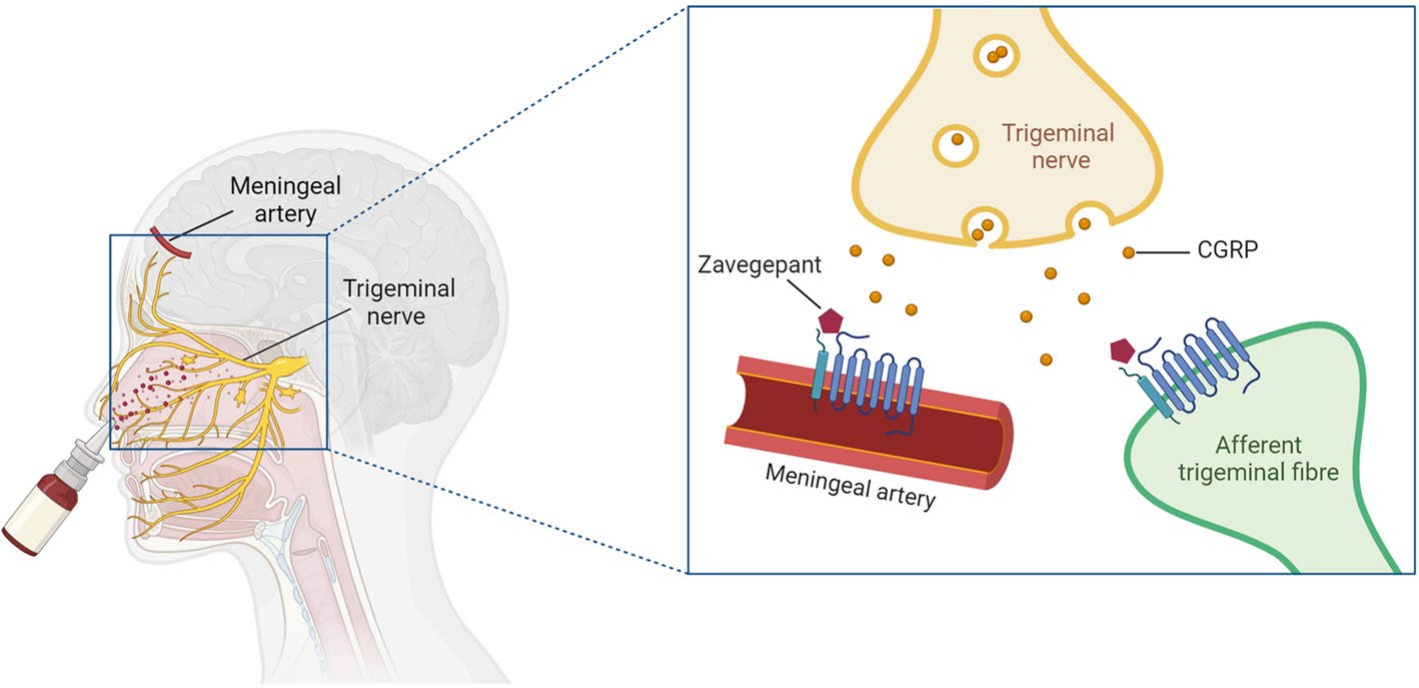
Local delivery of zavegepant directly to the trigeminovascular system through intranasal administration may improve pharmacokinetics, in addition to systemic absorption via the nasal mucosa. The nasal cavity is largely lined with trigeminal nerve endings. Created with BioRender.com

The similar efficacy of zavegepant may indicate that its intranasal administration has not only a systemic effect, but rather a local effect. Indeed, the findings from the current study corroborate this hypothesis. Trigeminal nerve endings innervate the HMMA, which renders it a good proxy for the trigeminovascular system [35]. The nasal cavity is also largely lined with trigeminal nerve endings [35]. In HMMA, we show here that zavegepant has a relatively low potency to block CGRP-induced relaxation at its therapeutic plasma concentration (i.e., C_max_). Therefore, these findings may indicate the relevance of local delivery directly to the trigeminovascular system through intranasal administration (Fig. 4).

The trigeminal nerve innervates both the HMMA and nasal cavity, but not via the same afferents. The HMMA is mainly, but not exclusively, innervated by the ophthalmic branch (V1), whereas the trigeminal nerve endings lining the nasal cavity are from the maxillary branch (V2) [20, 35]. Previous research has shown that activation of different branches of the trigeminal nerve is relevant in migraine, which may demonstrate general trigeminal activation during a migraine attack. CGRP measured in saliva (mandibular branch/V3) is related to the different migraine phases [3] and has shown promise as a predictor for therapeutic responses to rizatriptan [15] and erenumab [2]. In the forehead model (V1) CGRP-mediated trigeminovascular reactivity is related to the treatment response of erenumab [17]. During a migraine attack, accompanying symptoms such as nasal congestion and rhinorrhoea are present in some migraine patients [5], which may hint at activation of V2. In addition, V1 is not the only branch supplying the dura mater; collaterals from V2 and V3 also provide dural innervation to small regions [20]. Future research should investigate the exact role of each part of the trigeminal nerve during a migraine attack.

If zavegepant is indeed directly delivered to the trigeminovascular system, this might have additional advantages. As it is expected that gepants distribute to adipose tissues given their lipophilicity, their plasma concentrations would be lower in overweight patients with migraine, possibly leading to reduced efficacy [14]. However, if zavegepant is indeed delivered directly to the trigeminal nerve endings, this could be beneficial for this group of patients [1]. One of the current questions concerning gepants, is which gepant will benefit which group of patients. Although evidence on the effect of sex, body mass index, or genetic background on the efficacy or side effects of gepants is lacking, it is known that results from the trials cannot be generalized to all patient populations [1]. Whether local delivery directly to the trigeminovascular system may circumvent pharmacokinetic differences between these patient populations has yet to be revealed.

In theory, intranasal delivery of zavegepant could not only be beneficial, but affect the nasal mucosa. CGRP induces vasodilation of the nasal mucosa, but the exact role of this functional response in sickness and health is not known [7, 39]. The zavegepant clinical trials have shown side effects including taste disorder, nasal discomfort, and throat irritation, but no nasal congestion or mucosal disorder have been reported thus far [36]. Real-world studies are needed to assess whether intranasal delivery of zavegepant induces long-term nasal side effects.

Despite ongoing debate on whether the main effect of gepants is peripheral or central, our current analysis underscores the importance of their peripheral action. Although some research suggests that intranasal delivery allows medications to bypass the blood–brain barrier via the olfactory nerve [23, 31, 34, 41], this has not been demonstrated for the gepants. Localization of gepants has been observed at the trigeminovascular system and dura, which fall outside the protection of the blood–brain barrier [22]. A minor extent of blood–brain barrier crossing (1.4%) has been revealed for oral telcagepant in rhesus monkeys [48]. Although evidence is lacking, it is likely that these results are generalizable for all gepants. As estimated from our current analysis, free fraction concentrations of gepants in the central nervous system are likely in the picomolar range, which is insufficient to potently block CGRP-induced functional responses. Considering the abundant expression of CGRP in the central nervous system and the absence of reported central side effects with any gepant, this further suggests that the medication class primarily exerts its action at a peripheral site [13, 32].

This study has several limitations. First, even though our calculations are based on large amounts of data, all calculations can only approximate in vitro results. These calculations are rather a way to extract more value from available data without using large amounts of scarce material. Our comparison is meant to make a general comparison and to be hypothesis-generating. The clinical data being reported in many different statistical formats and being based on studies with a low number of participants also reduces the accuracy of the comparison between gepants. Second, data from the clinical studies on pharmacokinetics were mainly obtained from healthy volunteers participating in phase 1 trials, and the sex and body mass index of participants were not mentioned in every publication. Responses to CGRP might be different in patients with migraine due to a potentially altered balance and expression of CGRP and its receptors in the trigeminovascular system as compared with controls.

## Conclusion

In conclusion, the present study applied a novel method to connect pharmacodynamics and pharmacokinetics of gepants by combining data from clinical and basic research. Using this hypothesis-generating approach, we show that all gepants are effective at inhibiting functional responses to CGRP at their therapeutic plasma levels. The relatively low predicted potency of zavegepant to inhibit CGRP-induced relaxation at its therapeutic systemic plasma concentration may point to the relevance of local delivery to the trigeminovascular system through intranasal delivery, which should be further investigated in future studies.

### Supplementary Information

Supplementary Material 1.

## Data Availability

The datasets used and/or analysed during the current study are available from the corresponding author on reasonable request.

## Abbreviations

CGRP: Calcitonin gene-related peptide
C_max_: Maximum plasma concentration
HMMA: Human middle meningeal artery
pA_2_: The negative logarithm of the molar concentration that produces a twofold shift to the right in the agonist dose–response curve
pEC_50_: Negative logarithm of the molar concentration of an agonist that would induce half of the maximum response
pK_B_: The negative logarithm of the molar concentration that would occupy 50% of the receptors at equilibrium
