# Supplementary material for: Comparison of gepant effects at therapeutic plasma concentrations: connecting pharmacodynamics and pharmacokinetics
Source: J Headache Pain. 2024 Aug 28;25(1):141. doi: 10.1186/s10194-024-01846-8 (PMC11351853; doi:10.1186/s10194-024-01846-8)
Supplement: Supplementary file 1 — Supplementary Material 1. [file 10194_2024_1846_MOESM1_ESM.docx]

# Supplemental material

**Supplemental Table 1.** Overview of data obtained from literature on the pharmacological characterization of gepants in human middle meningeal arteries. pK_B_ values are based on reported pEC_50_ values for 10 nM of a gepant in HMMA.

| Gepant | Control pEC_50 ­_  (mean ± SEM) | Potency |
| --- | --- | --- |
| Rimegepant  (Mulder et al., 2020) | 8.22 ± 0.15 | 10.02  (pA_2_) |
| Ubrogepant  (Rubio-Beltran et al., 2020) | 8.38 ± 0.15 | 9.70  (pK_B_) |
| Atogepant  (Rubio-Beltran et al., 2020) | 8.38 ± 0.15 | 10.96  (pK_B_) |
| Zavegepant  (This article) | 8.40 ± 0.09 | 10.02 ± 0.07  (pK_B_) |

pA_2_, the negative logarithm of the molar concentration that produces a 2-fold shift to the right in the agonist dose-response curve; pEC_50_, negative logarithm of the molar concentration of an agonist that would induce half of the maximum response; pK_B_, the negative logarithm of the molar concentration that would occupy 50% of the receptors at equilibrium.
